# Additive quantile mixed effects modelling with application to longitudinal CD4 count data

**DOI:** 10.1038/s41598-021-97114-9

**Published:** 2021-09-09

**Authors:** Ashenafi A. Yirga, Sileshi F. Melesse, Henry G. Mwambi, Dawit G. Ayele

**Affiliations:** 1 School of Mathematics, Statistics, and Computer Science, University of KwaZulu-Natal, Pietermaritzburg, Private Bag X01, Scottsville, 3209 South Africa; 2 Institute of Human Virology, School of Medicine, University of Maryland, Baltimore, MD 21201 USA

**Keywords:** Biomarkers, Prognostic markers, HIV infections

## Abstract

Quantile regression offers an invaluable tool to discern effects that would be missed by other conventional regression models, which are solely based on modeling conditional mean. Quantile regression for mixed-effects models has become practical for longitudinal data analysis due to the recent computational advances and the ready availability of efficient linear programming algorithms. Recently, quantile regression has also been extended to additive mixed-effects models, providing an efficient and flexible framework for nonparametric as well as parametric longitudinal forms of data analysis focused on features of the outcome beyond its central tendency. This study applies the additive quantile mixed model to analyze the longitudinal CD4 count of HIV-infected patients enrolled in a follow-up study at the Centre of the AIDS Programme of Research in South Africa. The objective of the study is to justify how the procedure developed can obtain robust nonlinear and linear effects at different conditional distribution locations. With respect to time and baseline BMI effect, the study shows a significant nonlinear effect on CD4 count across all fitted quantiles. Furthermore, across all fitted quantiles, the effect of the parametric covariates of baseline viral load, place of residence, and the number of sexual partners was found to be major significant factors on the progression of patients’ CD4 count who had been initiated on the Highly Active Antiretroviral Therapy study.

Parametric models relate the mean of a response variable to a linear combination of covariate effects and focus on the response’s average properties^1^. Nevertheless, there are inevitable occasions when such parametric models fail, and data analysis must turn to more flexible, nonparametric models^2^. Parametric models also assume a distribution for the outcome variable as opposed to purely nonparametric models. However, most of the vast literature on nonparametric regression also deals with the estimation of conditional mean models. In addition, the conventional assumption of nonparametric regression theory that there is additive, independently, and identically distributed (*iid*) error around a smooth underlying conditional mean function is highly implausible in certain data settings^2^. Thus, as in the parametric context, nonparametric methods are usefully complemented by nonlinear estimation of families of conditional quantile functions that relax the independence assumption^2^. The use of parametric and nonparametric regression models for analyzing patients’ CD4 count in most applications implies that the estimated effects describe the average CD4 count. However, it is of even great interest to examine the quantile of the outcome distribution, such as the lower ($\le$ 25%) quantile, which identifies patients at higher risk of developing illnesses.

Quantiles, commonly symbolized by the Greek letter $\tau$, are location and scale parameters simultaneously. For a given $\tau \in \left( {0, 1} \right)$, the $\tau^{th}$ quantile is the value of a random variable, where $\tau \times 100\%$ of its value lies below it. In other words, it is the value where at most $\left( {1 - \tau } \right) \times 100\%$ of the value lies above. Thus, $\tau {\text{th}}$ quantiles close to 0.5-quantile give the median, which is a well-known location parameter. On the other hand, $\tau {\text{th}}$ quantiles close to zero or one give an idea of the scale. For instance, the interquartile range (IQR) is defined as the 0.75 quantile minus the 0.25 quantile: $IQR = Q_{3} - Q_{1}$.

Quantile regression (QR) solutions are computed for a selected number of quantiles, typically the three quantiles along with two extreme quantiles, that is, for $\tau  = \{ 0.05,0.25(Q_{1} ),0.5\left( {Q_{2} } \right),0.75\left( {Q_{3} } \right),0.95\}$. This necessitates the search for a suitable compromise between the amount of output to manage and the results to interpret and summarize. Although in many practical applications of QR, the focus is on estimating a subset of quantiles, however, it is worth noticing that it is possible to attain estimates across the entire interval of conditional quantiles; in particular, the set: $\left\{ {\beta_{\tau } :\tau \in \left( {0, 1} \right)} \right\}$^2^.

QR is a versatile statistical method with many applications that complement mean regression^3,4^. Thus, it emerged as an effective analytic technique in numerous study areas of science due to its competence to drive inferences about individuals that rank below or above the conditional population mean and/or focused on features of the response beyond its central tendency^4–13^. QR is specifically appropriate for the parameters' heterogeneous effect as it yields inferences that can be legitimate irrespective of the true underlying distribution^4,14^. QR techniques look further into the data, get more information, and become more important^15^. By fitting models for more percentiles, one can detect the covariates' heterogeneous effects at the conditional distribution of the response, rather than just the conditional mean. That is especially useful when valuable information lies at the bottom or top quantiles. “QR also enjoys several properties, including equivariance to monotone transformations and robustness to outliers”^2,16^. A semiparametric extension of quantile regression models with different types of nonlinear effects included in the model equation leads to an additive quantile regression model (AQM)^12^. Such a model may reveal systematic differences in dispersion, tail behavior, and other features for covariates^2^.

Additive mixed models (AMMs), an extension of additive models, have been developed precisely to incorporate linear and nonlinear effects, as well as random terms when the data are sampled according to longitudinal designs^4,17^. AMMs have been integrated into QR methods to obtain robust results, not only focused on features of the longitudinal outcome at its central tendency that may not be the best location to characterize the data specifically when the errors are non-normally distributed, and the location-shift hypothesis of the normal model is violated but also at conditional quantiles of the longitudinal outcome with no assumption about the response or errors distribution apart from the distribution is restricted to have the $\tau {\text{th}}$ quantile to be zero. Thus, additive quantile mixed models, which have gained popularity recently as a general method for longitudinal data, bring a comprehensive and more complete picture of the nonparametric as well as the parametric effects^1,4^.

CD4 cell count levels signify the well-being of an individual immune system (body’s natural defense system against pathogens, infections, and illnesses). The CD4 cell counts of a person who does not have HIV can be between 500 and 1500 per cubic millimeter. Individuals living with HIV who have a CD4 count over 500 but whose immune response is still strong are usually in good health. However, individuals living with HIV who have a CD4 count below 200 are at high risk of developing severe illnesses and death^18,19^.

With the CD4 count at deficient levels, patients’ immunity is weak. If HIV-infected patients are not on treatment or not virally suppressed, they become vulnerable to acquire opportunistic infections (OIs), making them at risk of the new and ongoing coronavirus disease 2019 (COVID-19) infection and underlying illness^18^. The best strategy to avoid these infections and diseases is by enhancing the immune function level through HAART, a combination of multiple antiretroviral (ARV) drugs. HAART’s fundamental goal is to prolong or stop the progression to AIDS and loss of life for those infected with HIV by suppressing and preventing the virus from making copies of itself. When the virus’s level (viral load) in the blood is low or undetectable, there is less damage to the body’s immune system and fewer HIV infection complications. Even though HIV treatment is prescribed for all individuals living with HIV, it is particularly critical for patients with low CD4 count to start treatment sooner rather than later and adhere to the treatment schedule^18,20^. While researchers believe that early diagnosis and effective treatment are essential to effective control, more research is needed to understand better the adaptive, innate, and host responses that alter viral load set-point and consequently prognosis and infectiousness^18,20^.

The need for good and better health is one of each human being’s fundamental rights without qualification of race, religion, gender, political conviction, financial, or social condition. Women’s health includes their emotional, social, and physical welfare and is determined by these factors and the economic setting of their lives, as well as by biology. However, health issues evade the longer part of women. In national and universal forums, women have emphasized that equality, the sharing of family duties, development, and peace are necessary conditions to achieve good health all through the life cycle. Women are biologically and socially more vulnerable to HIV infection, especially in developing countries^21–24^.

HIV/AIDS and other sexually transmitted diseases (STD) have a devastating effect on women’s health, mostly young ladies. The consequences of HIV/AIDS go beyond women’s health to include their families’ economic support and livelihoods. Thus, the social, development, and health consequences of HIV/AIDS and other sexually transmitted diseases have strong gender dimensions that cannot be ignored^23–25^. Understanding the changing epidemiology of HIV using statistical disease models will allow the clinician to decide who may be at high risk and clarify the application of rules to avoid sequential HIV transmission^18,20,26,27^. Although antiretroviral (ARV) recommendation presently remains the same for all individuals living with HIV, examining the progression of CD4 count or evolution of the viral load using data-driven models will allow the clinician to interpret potential information accurately and cope with misdirection or distortion of the information due to patient-specific effects^18,26–28^. This study is a continuation of our previous work in Yirga et al.^18^. This study aims to analyze the longitudinal CD4 count of HIV-infected patients involved in a CAPRISA study using AQMM and justify how the method evolved can be used to attain robust nonparametric as well as parametric effects at various locations of the conditional distribution that brings a comprehensive and more complete picture of the covariate effects. The use of AQMM has many advantages. Additive nonparametric effects models are not new in the applied statistics literature. To implement these methods, Koenker et al.^47^ introduce smoothing penalties for total variation, especially for the nonparametric components of the model. Researchers are also eager to learn what are the factors influencing the CD4 count (high or low) in HIV studies. AQMMs are the best way to answer this question.

## Materials and methods

### Data description

This study used data from the Centre for the AIDS Programme of Research in South Africa (CAPRISA). The CAPRISA study was effected at the Doris Duke Medical Research Institute (DDMRI) at the Nelson R Mandela School of Medicine of the University of KwaZulu-Natal in Durban, South Africa^18,29^. Between August 2004 and May 2005, CAPRISA introduced a cohort study registering high-risk HIV-negative women to a follow-up study with an intense ongoing examination. Women infected with HIV were recruited into the CAPRISA 002 Acute Infection (AI) study and then followed up carefully to study disease progression and CD4/viral load evolution^18,20,29–32^.

**Figure 1:**
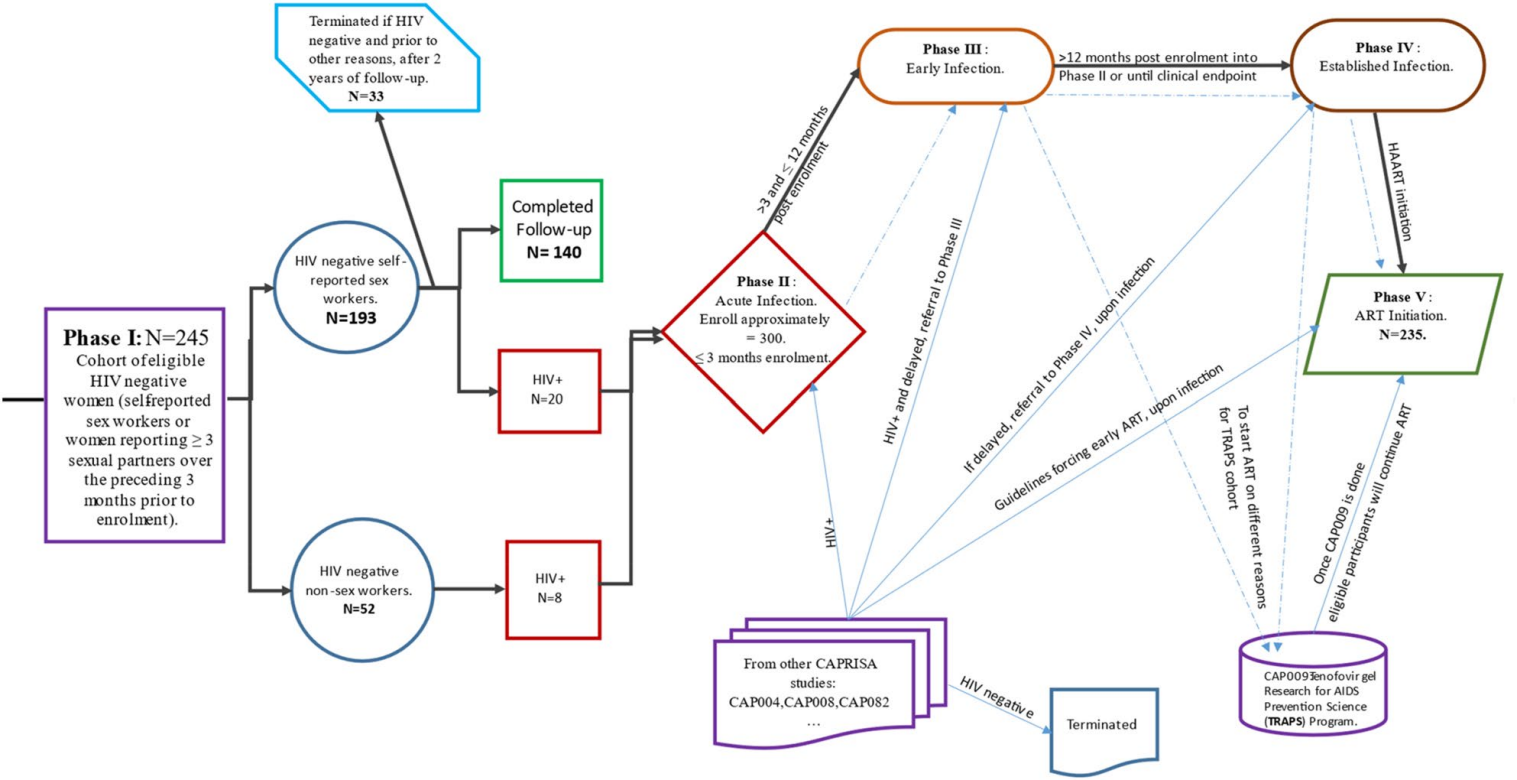
Diagrammatic overview of the CAPRISA 002 AI cohort study design.

Once HIV-infected women were enrolled in CAPRISA’s AI Phase II study, their CD4 count and viral load were measured and assessed regularly. When their CD4 count $\le 350$ cells/mm^3^ for more than two consecutive visits between six months or if they are with AIDS-defining illness (WHO clinical stage 3–5), they would be referred to a public government clinic for ARV treatment. However, according to the South African National Department of Health, these patients would only start HAART once their CD4 count is $\le 200$ cells/mm^3^, until 2015. With effect from the 1st of January 2015, according to the National Department of Health, the criteria to start HIV patients on early initiation of ART is CD4 count of $500$ cells/mm^3^ or less than that^20^. HIV-infected women in Phase II–IV were followed up until they are started HAART. After that, they would be transitioned to Phase V and followed up for a minimum of five years, or eligible participants would be offered to join immediately into Phase V^33^. After the five years of follow-up have been accomplished, participants would be offered an optional annual follow-up for up to fifteen extra years to patients who recurred in Phase V^33^. Figure 1 illustrates the screening and enrolment process of the study data set. One can find further detail on the study population’s design, development, and procedures here^29–33^.

### Consent for publication

Not applicable.

## Methods

Parametric regression models typically use a linear function to connect the conditional values of the response variable to the covariates. In real-world applications, however, biased or invalid results might result from such a linearity assumption. Many studies use nonlinear assumptions between variables^34–37^. One may consider various modeling techniques when dealing with nonlinearity. The most popular nonparametric models, smoothing splines, and transformation models use parameters such as sampling designs (cross-sectional or longitudinal), outcomes (discrete or continuous), distribution assumptions (parametric or nonparametric), and so on^2^. In choosing which method to follow, the amount of effort expended during the investigation may have a significant influence. Likewise, lacking theory or programming can lead to a certain decision being made over another^2^.

Nonparametric regression permits the presumption of linearity to be relaxed^34,35,38^ and limits the analysis to smooth and continuous functions^39^. Nonparametric regression, also known as scatter smoothing, aims to distinguish the best regression function according to the data distribution instead of estimating the parameters^39^.

The nonparametric regression model is given by.

1 $$ y = \mathop \sum \limits_{i = 1}^{n} f_{i} \left( {x_{i} } \right) + \varepsilon_{i}, $$

where the function $f_{i} \left( \cdot \right)$ is unknown, and commonly assumed that the errors are normally and identically distributed: $\varepsilon_{i} \sim NID\left( {0, \sigma^{2} } \right)$^39^. Several methods have been introduced to model nonparametric regression models; however, the most used techniques that have been extended to QR are local polynomial regression^40^ and smoothing splines^41,42^: for further details, see Wu and Zhang^34^, Fox^38^, Davino et al.^39^, Craig and Ng^43^, Koenker et al.^44^, Koenker^45^, Cleveland and Loader^46^, or Koenker et al.^47^.

The parametric QR model is given by.

2 $$ Y_{i} = {\varvec{x}}_{i}^{^{\prime}} {\varvec{\beta}}_{\tau i} + \varepsilon_{\tau i} , \quad i = 1, \ldots ,n, \quad 0 < \tau < 1, $$

where $Y_{i}$ is the response variable, $x_{i}$’s are covariates, $\beta_{\tau i}$’s are the quantile specific linear effects, and $\varepsilon_{\tau i}$ is a random variable assumed to be an unknown error term on which no specific distributional assumptions are made except that the distribution is restricted to have the $\tau {\text{th}}$ quantile to be zero^12,48,49^. For this reason, the parametric QR model aims at describing the quantile function $Q_{{Y_{i} }} \left( {\tau |{\varvec{x}}_{i} } \right)$ of the continuous outcome $Y_{i}$ conditional on covariate vector ${\varvec{x}}_{i}$ at a given quantile $\tau$, and this can be expressed as follows

3 $$ Q_{{Y_{i} }} \left( {\tau |{\varvec{x}}_{i} } \right) = F_{{Y_{i} }}^{ - 1} \left( {\tau |{\varvec{x}}_{i} } \right) = {\varvec{x}}_{i}^{^{\prime}} {\varvec{\beta}}_{\tau i} + \varepsilon_{\tau i}, \quad {\text{with}}\;Q_{{\varepsilon_{\tau i} }} \left( {\tau |{\varvec{x}}_{i} } \right)\sim F_{\tau i} , $$

where $F_{\tau i}$ is subject to $F_{\tau i} \left( 0 \right) = \tau$, $F_{{Y_{i} }}^{ - 1} \left( \cdot \right)$ is the inverse cumulative distribution function of $Y_{i}$. For a comprehensive overview of QR, see, for example, Koenker^2^, Konker and Basset^3^, Buchinsky^5^, Yu et al.^9^, or Koenker and Hallock^50^.

As much as the parametric QR assumptions enjoy a simple model structure, convenience of interpretation, and lower computational cost, it is not flexible enough and hence carries the risk of model misidentifications for complex problems^51^. Nonparametric QR has become a viable alternative to avoid restrictive parametric assumptions. Koenker et al.^47^ explored nonparametric QR in spline models (quantile smoothing splines), which they defined as solutions to4where $\rho_{\tau } \left( u \right) = u\left\{ {\tau - I(u < 0)} \right\}, p \ge 1$, is the so-called *check (loss) function,* the parameter $\tau \in \left( {0, 1} \right)$ controls the quantile of interest, and $\lambda \in {\mathbb{R}}^{ + }$ is a smoothing parameter^3,47^.

As closely analogous to the parametric QR model (3), Koenker^2^ generalized nonparametric QR models as

5 $$ Q_{{Y_{i} }} \left( {\tau |{\varvec{x}}_{i} } \right) = f\left( {{\varvec{x}}_{i} ,\beta_{i} \left( \tau \right)} \right) $$

Then, Koenker^2^ formulated the $\tau {\text{th}}$ nonparametric QR estimator as

6 $$ \hat{\user2{\beta }}_{{\varvec{i}}} \left( \tau \right) = \mathop {{\text{argmin}}}\limits_{\beta } \mathop \sum \limits_{i = 1}^{n} \rho_{\tau } \left( {y_{i} - f\left( {{\varvec{x}}_{i} ,\beta \left( \tau \right)} \right)} \right) $$

Several techniques were proposed for nonparametric QR modelings, such as Bivariate quantile smoothing spline^52^ and Kernel quantile regression^53^. However, nonparametric QR is an important yet challenging topic that needs to be addressed in-depth^51^. One can find a brief account of nonparametric QR strategies in numerous studies; see, for example, Koenker^2^ or Davino et al.^39^. To account for the nonlinearity relationships between quantiles of the outcome and covariates, Rigby and Stasinopoulos^54^ also proposed generalized additive models for location, scale, and shape (GAMLSS). GAMLSS enables additional flexibility to fit the covariates' nonlinear effects; however, they do not result in easily interpretable expressions for the quantiles. They are based on specifying distinct distributional parameters^12^. Instead, additive quantile regression models (AQMs) allow for the inclusion of nonlinear covariate effects and give more flexibility^12^.

Additive models, introduced by Hastie and Tibshirani^41^, Stone^55^, and Breiman and Friedman^56^, are flexible regression tools that manipulate linear as well as nonlinear terms. The nonlinear terms in additive models are modeled through smoothing splines^4^. They provide programmatic approaches for nonparametric (nonlinear in parameters) regression modelings; by restricting nonlinear covariate effects to be composed of low-dimensional additive pieces so that we can overcome some of the worst aspects of the notorious curse of dimensionality^11^. The literature on additive models is vast^17,41,55,57,58^. However, most of the work has been done based on estimating conditional mean functions. The additive quantile regression model (AQM) provides an attractive framework for parametric as well as nonparametric regression illustrations focused on features of the response beyond its central tendency^4,11,12^.

Fenske et al.^12^ defined the $\tau {\text{th}}$ AQMs that extend the linear predictor, ${\varvec{x}}_{i}^{^{\prime}} {\varvec{\beta}}_{\tau }$, with a sum of nonlinear functions of continuous covariates, $\sum f_{\tau j} \left( \cdot \right)$, as follows.

7 $$ Q_{{Y_{i} }} \left( {\tau |{\varvec{x}}_{i} ,{\varvec{z}}_{i} } \right) = {\varvec{x}}_{i}^{^{\prime}} {\varvec{\beta}}_{\tau i} + \mathop \sum \limits_{j = 1}^{q} f_{\tau j} \left( {{\varvec{z}}_{i} } \right) + \varepsilon_{\tau i} , \quad j = 1, \ldots ,q, $$

where $f_{\tau j}$ denote generic functions of covariates ${\varvec{z}}_{i}$ for the $i{\text{th}}$ observation, and allows for the inclusion of different model terms such as nonlinear effects (smooth functions) of $z_{k}$, $f_{\tau } \left( {z_{k} } \right)$, and varying coefficient terms, $z_{k}^{^{\prime}} f_{\tau } \left( {z_{k} } \right)$, where the effect of the covariate $z_{k}^{^{\prime}}$ varies smoothly over the domain of $z_{k}$ according to some functions of $f_{\tau }$. However, the underlying assumption of the error term, $\varepsilon_{\tau i}$, remains the same as in the QR model (3); see Fenske et al.^12^ for more details.

AQM estimates the additive effect using linear programming algorithms as in the conventional QR model^12^. However, in the AQM case, determining adequate numbers and the position of knots is challenging. To avoid these challenges, Fenske et al.^12^ used penalty methods such as quantile smoothing splines of Koenker et al.^47^. Thus, the minimization problem of AQM that consists of extra penalty term is given by^12^:

8 $$ \mathop {{\text{argmin}}}\limits_{{f_{\tau } }} \mathop \sum \limits_{i = 1}^{n} \rho_{\tau } \left( {y_{i} - {\varvec{x}}_{i}^{^{\prime}} {\varvec{\beta}}_{\tau i} - \mathop \sum \limits_{j = 1}^{q} f_{\tau j} \left( {{\varvec{z}}_{i} } \right)} \right) - \lambda {\varvec{v}}\left( {f_{\tau }^{^{\prime}} } \right), $$

where ${\varvec{v}}\left( {f_{\tau }^{^{\prime}} } \right) = sup\mathop \sum \limits_{i = 1}^{n - 1} \left| {f_{\tau }^{^{\prime}} \left( {z_{i + 1} } \right) - f_{\tau }^{^{\prime}} \left( {z_{i} } \right)} \right|$, represents the total variation of the derivation $f_{\tau }^{^{\prime}} :\left[ {a, b} \right] \to {\mathbb{R}}$, where the $sup$ is taken over all partitions $a \le z_{1} < \ldots < z_{n} < b$, and $\lambda$ is a tuning parameter that controls the smoothness of the estimated function also known as “total variation regularization”: see Koenker^2^, Fenske et al.^12^, or Koenker et al.^47^ for more details.

Fenske et al.^1^ proposed extending AMMs to the QR model for longitudinal data that consists of fixed individual-specific intercepts and slopes modeled through penalized splines of Ruppert et al.^59^. However, their model did not include random-effect terms and did not allow for individual-specific effects to have a general covariance structure^4^. The version of Geraci^4^ additive QR model for longitudinal data includes linear and nonlinear terms, as well as multiple random effects to account for the correlation at the individual level with a general variance–covariance matrix and allow for automatic smoothing selection within a mixed model framework of Ruppert et al.^59^. Thus, as pointed out by Geraci^4^, because of the following two basic ideas, his model was shown to have superior performance compared with the approach of Fenske et al.^1^: the first point is regarding the $i{\text{th}}$ unit effects, which he assumed to be random instead of fixed so that the covariance structure between effects can be introduced; the second point is that instead of prior specification, the nonparametric term's smoothing is automatically estimated from the data^4^.

Geraci^4^ defined the $\tau {\text{th}}$ additive QR model for longitudinal data as

9 $$ Q_{{y_{ij} |{\varvec{u}}_{i} ,{\varvec{x}}_{i} ,{\varvec{z}}_{i} }} \left( \tau \right) = \beta_{\tau ,0} + \mathop \sum \limits_{k = 1}^{p} f_{\tau }^{k} \left( {x_{ijk} } \right) + z_{ij}^{^{\prime}} u_{\tau ,i} , $$

$$ j = 1, \ldots , n_{i} , \quad i = 1, \ldots ,m, \quad \tau \in \left( {0, 1} \right), $$

where $x_{ij}^{^{\prime}}$ is the $j{\text{th}}$ row of a known $n_{i} \times p$ matrix ${\varvec{X}}_{i}$, $z_{ij}^{^{\prime}}$ is the $j{\text{th}}$ row of a known $n_{i} \times q$ matrix ${\varvec{Z}}_{i}$, $y_{ij}$ is the $j{\text{th}}$ observation of the response vector ${\varvec{y}}_{i} = \left( {y_{11} , \ldots , y_{{1n_{i} }} } \right)^{^{\prime}}$ for the $i{\text{th}}$ unit, $f_{\tau }^{k} \left( \cdot \right)$ is a $\tau$-specific, centered, twice-differentiable smooth function of the $k{\text{th}}$ component of ${\varvec{x}}$, and $u_{\tau ,i}$ is a $q \times 1$ vector of values that collects $i{\text{th}}$ unit random effects associated with $z_{ij}$ and its distribution is assumed to depend on a $\tau$-specific parameter^4^.

Geraci^4^ considered a spline model of the type: $f_{\tau } \left( x \right) \approx \sum\nolimits_{h = 1}^{H} {v_{\tau } } ,_{ h} B_{ h} \left( x \right)$, to model nonlinear functions of the components of ${\varvec{x}} = (x_{1} , \ldots , x_{s} ,x_{s + 1} , \ldots ,x_{p} )^{^{\prime}}$ that consists of the first $s$ terms of nonlinear functions and $p - s$ linear functions. The $B_{h}$’s denote the *basis functions* ($v_{\tau }$), $h$’s represent the corresponding $\tau$-specific coefficients of $B_{h}$’s and $H$ indicates the number of knots^4^. The approximated quantile function from the model (9) is then expressed as follows^4^:

10 $$ Q_{{y_{ij} |{\varvec{u}}_{i} ,{\varvec{x}}_{i} ,{\varvec{z}}_{i} }}^{*} \left( \tau \right) = \beta_{\tau ,0} + \mathop \sum \limits_{k = 1}^{s} \mathop \sum \limits_{h = 1}^{{H_{k} }} v_{\tau } ,_{ hk} B_{ h}^{\left( k \right)} \left( {x_{ijk} } \right) + \mathop \sum \limits_{k = s + 1}^{p} \beta_{\tau } , _{k} x_{ijk} + z_{ij}^{^{\prime}} u_{\tau ,i} $$

In matrix notation, the $i{\text{th}}$ unit of expression (10), which is then called additive quantile mixed model (AQMM), is given by^4^

11 $$ Q_{{{\varvec{y}}_{i} |{\varvec{u}}_{i} ,{\varvec{x}}_{i} ,{\varvec{z}}_{i} }}^{*} \left( \tau \right) = {\varvec{F}}_{i} {\varvec{\beta}}_{\tau } + {\varvec{Z}}_{i} {\varvec{u}}_{\tau ,i} + {\varvec{B}}_{i} {\varvec{v}}_{\tau } , $$

where $B^{\left( k \right)} \left( {x_{ijk} } \right)$ is considered as $H_{k \times 1}$ vector of values taken by the $k{\text{th}}$ spline evaluated at $x_{ijk}$, $v_{\tau , k} = (v_{\tau , 1} , \ldots , v_{{\tau ,H_{k} }} )^{^{\prime}}$ considered as the $H_{k \times 1}$ vector of spline coefficients for the $k{\text{th}}$ covariate, and $H = \mathop \sum \limits_{k} H_{k}$. Furthermore, ${\varvec{B}}_{i}$ and ${\varvec{v}}_{\tau }$, defined, respectively, as the $n_{i} \times H$ matrix with rows $\left( {B^{\left( 1 \right)} \left( {x_{ij1} } \right)^{^{\prime}} , \ldots ,B^{\left( s \right)} \left( {x_{ijs} } \right)^{^{\prime}} } \right)^{^{\prime}}$ and $\left( {v_{\tau ,1}^{^{\prime}} , \ldots , v_{\tau ,s}^{^{\prime}} } \right)^{^{\prime}}$, ${\varvec{F}}_{i}$ is the $n_{i} \times \left( {p - s + 1} \right)$ matrix with rows $\left( {1, x_{{ij\left( {s + 1} \right)}} , \ldots , x_{ijp} } \right)^{\prime}$ and ${\varvec{\beta}}_{\tau } = \left( {\beta_{\tau ,0} , \beta_{\tau ,s + 1} , \ldots , \beta_{\tau ,p} } \right)^{\prime}$^4^.

The objective function of AQMM, where the vectors ${\varvec{u}}_{\tau ,i}$ and ${\varvec{v}}_{\tau }$ are assumed to follow zero-centered multivariate Gaussian distributions with variance–covariance matrices ${\varvec{\varSigma}}_{\tau }$ and ${\varvec{\varPhi}}_{\tau } = \oplus_{k = 1}^{s} \phi_{\tau } ,_{ k} I_{{H_{k} }}$, respectively, with selecting $\rho_{\tau } \left( {\varvec{r}} \right) = \mathop \sum \limits_{j = 1}^{n} r_{j} \left\{ {\tau - I\left( {r_{j} < 0} \right)} \right\}$ for a vector ${\varvec{r}}$ = $\left( {r_{1} , \ldots , r_{n} } \right)^{^{\prime}}$, is given by Geraci^4^ as

12 $$ \mathop \sum \limits_{i = 1}^{M} \rho_{\tau } \left( {{\varvec{y}}_{i} - {\varvec{F}}_{i} {\varvec{\beta}}_{\tau } - {\varvec{Z}}_{i} {\varvec{u}}_{\tau ,i} - {\varvec{B}}_{i} {\varvec{v}}_{\tau } } \right) +  \mathop \sum\limits_{{i = 1}}^{M}|| {u_{{\tau ,i}}||^2_{\Sigma _{\tau }^{ - 1} }}  + \mathop \sum \limits_{k = 1}^{s} \phi_{\tau ,k}^{ - 1}|| v_{\tau , k}||^{2} , $$

where “${\varvec{u}}_{\tau ,i}$’s are assumed to be independent for different $i$ (but may have a general covariance matrix) and are independent of ${\varvec{v}}_{\tau }$, and $\phi_{\tau ,k}$’s determine the amount of smoothing for the nonparametric terms”^4^. Minimizing the objective function of expression (12) proceeds as the same as minimizing the objective function of quantile mixed-effects models^49,60,61^ where the asymmetric Laplace distribution with a location parameter $\mu$, scale parameter $\sigma > 0$, and skewness parameter $\tau \in \left( {0, 1} \right)$^60,62–64^, are employed as *quasi-likelihood* for the fidelity term^4^. Further discussion of AQMM is provided by Geraci^4^.

### Ethical approval and consent to participate

The study was approved by the Research Ethics Committee of the University of KwaZulu-Natal (E013/04), the University of the Witwatersrand (MM040202), and the University of Cape Town (025/2004). All participants provided written informed consent. All methods were performed following the relevant guidelines and regulations expressed in the Declaration of Helsinki.

## Results

Geraci^4^ illustrated the full range of AQMM that is described above. The purpose of this analysis is to model the CD4 count of patients from KwaZulu-Natal, South Africa, as part of a comprehensive study of HIV/AIDS. The results of this study illustrate longitudinal CD4 counts among HIV-infected patients enrolled in the CAPRISA 002 AI study by employing an AQMM. The median age of our sample of 235 women was 25 years. Our sample consisted of 7019 measurements on 235 women from 18 to 59 years of age. There were multiple visits for all participants, ranging from 2 to 61, with a median of 29.

**Figure 2:**
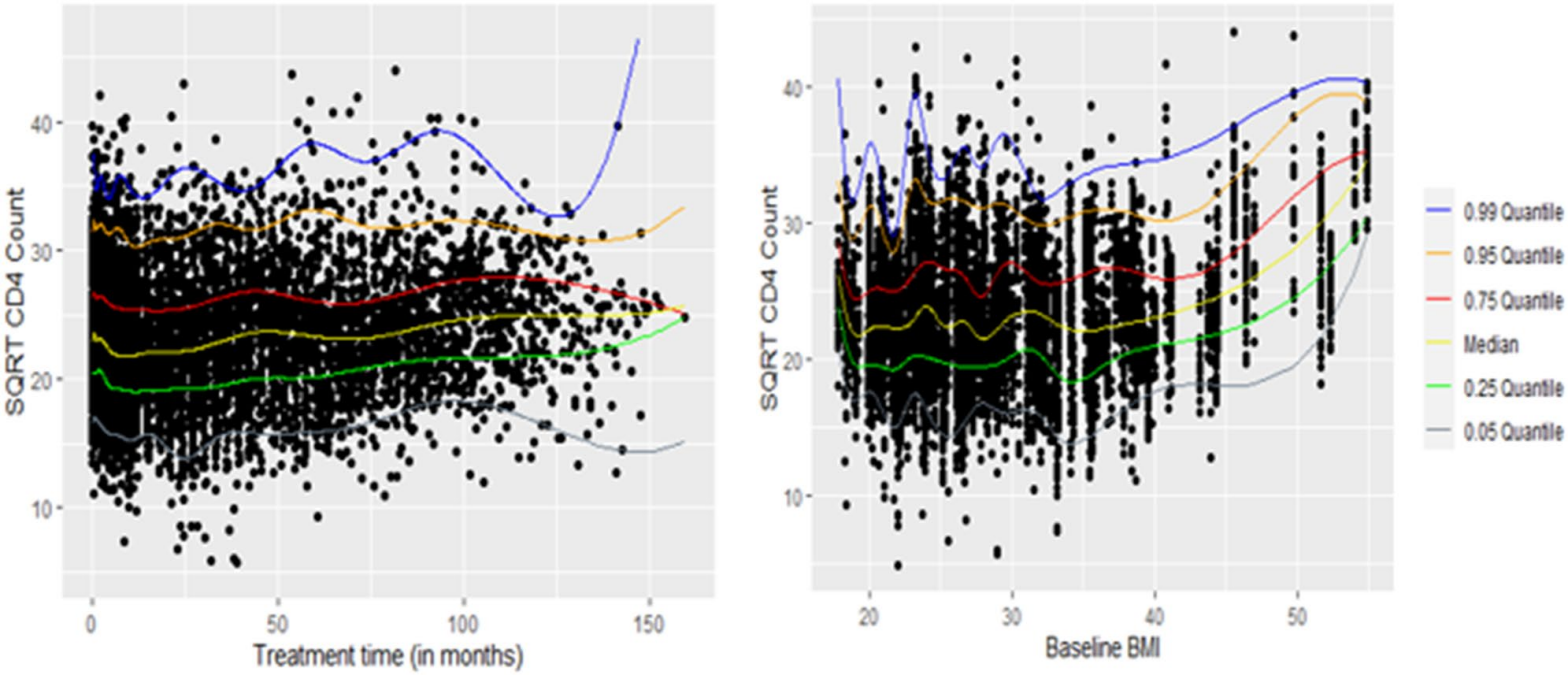
Observed CD4 counts (square root transformed) by time and baseline BMI.

Tables 1 and 2 show descriptive measures for the variables studied. Low (upper) quantiles are those where at least 25% (75%) of the observations are at or below it, or 75% (25%) are at or above it^2^. In Table 1, it is shown that the median BMI for the participants was 26.84 (range 17.89–54.89). The median square root CD4 count and baseline viral load were 22.98 cells/mm^3^ and 26,600 copies, respectively. Of a total of 235 women, 105 (44.7%) lived around Vulindlela (rural area), and 130 (55.3%) lived around eThekwini (Durban, urban area) in KwaZulu-Natal, South Africa (see Table 2). The majority of the women, 182 (77.4%), were in a stable partnership, 224 (95.3%) completed secondary school (Table 2), and most of them (78.8%) were self-reported sex workers^18,29,31^. Additional details are available here^29–32^ concerning the CAPRISA 002 AI study. We analyze this study data set intending to explain the different conditional distribution of the CD4 count by considering two covariates entered as nonparametric additive effects: time and baseline BMI; as well as discrete (baseline viral load), continuous (age), and categorical covariates (place of residence, educational level, and the number of sexual partners) entered in the model as parametric effects (see Tables 1, 2). Figure 2 shows observed square root transformed CD4 counts by treatment time and baseline BMI, respectively, for a total of 7019 observations. The nonlinear patterns, which connect the sample quantiles, are estimated conditionally on time and baseline BMI for six quantile levels. The curves (nonlinear patterns) suggest the requirement of some degree of smoothing (Fig. 2).

**Table 1:** Descriptive statistics for non-categorical variables.

Variable	Descriptive measures						
	Mean	Median	Minimum	Maximum	Q_0.25_	Q_0.75_	IQR
**SQRT_CD4 count (cells/µL)**	23.26	22.98	5	44	20	26.19	6.19
**Baseline VL (**cells/**mL)**	130,730.33	26,600	1 (undetected)	5,510,000	5080	113,000	107,920
**Age (Years)**	27.15	25	18	59	22	30	8
**Baseline BMI**	28.98	26.84	17.89	54.89	23.33	32.96	9.63

**Table 2:** Baseline descriptive statistics for categorical variables.

Variable	Total	Variable	Total
**Place of residence**		**Number of sexual partners**	
Rural (*reference*)	105 (44.7%)	No partner (*reference*)	43 (18.3%)
Urban	130 (55.3%)	Stable partner	182 (77.4%)
**Educational level**		Many partners	10 (4.3%)
Primary schools (*reference*)	11 (4.7%)	**Number of women**	235
Secondary schools	224 (95.3%)

Following the AQMM of Geraci^4^, we used a transformed continuous form of the outcome (i.e., square root CD4 count) for fitting purposes. Thus, the proposed $\tau^{th}$ AQMM form of our study, using expression (10), can be specified as

13 $$ \begin{aligned} Q_{{y_{ij} |{\varvec{u}}_{i} ,{\varvec{x}}_{i} ,{\varvec{z}}_{i} }}^{*} \left( \tau \right) & = \beta_{\tau ,0} + \mathop \sum \limits_{h = 1}^{{H_{1} }} v_{\tau } ,_{ 1} B_{ h}^{\left( 1 \right)} \left( {time_{i} } \right) + \mathop \sum \limits_{h = 1}^{{H_{2} }} v_{\tau } ,_{ 2} B_{ h}^{\left( 2 \right)} \left( {BMI_{i} } \right) + \beta_{\tau } ,_{ 1} ART_{i} \\ & \quad + \beta_{\tau } ,_{ 2} VL_{i} + \beta_{\tau } ,_{ 3} residence_{i} + \beta_{\tau } ,_{ 4} education_{i} + \beta_{\tau } ,_{ 5} partner_{i} \\ & \quad + \beta_{\tau } ,_{ 6} age_{i} + u_{\tau ,0} + u_{\tau ,1} \left( {time_{i} } \right), \\ \end{aligned} $$

where $y_{ij}$ is the square root transformed form of the outcome ($\sqrt {CD4} count$) at the $j{\text{th}}$ time point for the $i{\text{th}}$ subject, time is the time variable measured in months from the start of the study, BMI indicates the patient’s baseline BMI, ART is the dichotomous HAART initiation (0 = pre-ART, 1 = post-ART), VL is patient’s baseline viral load, the residence is patient’s place of residence, education is the educational level of participants, partner indicates the number of sexual partners of the participant, age is participant age at enrolment, $u_{\tau ,0}$ indicates the random intercept, and $u_{\tau ,1}$ indicates the random slope. The symbol $\tau$ specifies the quantile of interest; we made the estimation at $\tau = 0.05, 0.25, 0.5, 0.75, 0.85, 0.95,$ and $0.99$ to get the complete picture of the effects.

**Figure 3:**
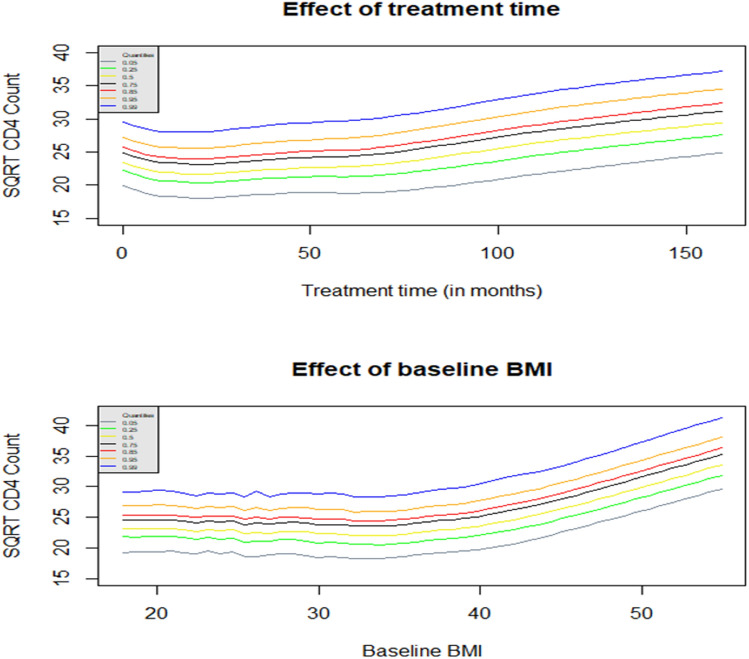
Predicted smoothed covariate effects on the square root CD4 count of HIV-infected patients recurred in the CAPRISA 002 AI study at various quantiles using AQMM.

Geraci^4^ employed the AQMM in the R package *lqmm* as an ad-on to fit additive quantile mixed models. As the same as the smooth terms’ specification in the R package *mgcv*^17^, one can enter continuous covariates within the *s* (smooth) function to control the model smoothness using splines when fitting AQMM^4^. Furthermore, the shrinkage smoothers obtained using the *bs* option inside the *s* command in the R package *mgcv* are constructed so that smooth terms can be penalized away altogether, not contribute to the model^17,65^. Thin plate smoother provides statistical and computational efficiency, stable optimal approximations (especially for large data sets), and can be constructed for smooths of more than one covariate at a time^4,66^. Thus, it was used as a shrinkage spline to fit the proposed model (13). The remaining parametric terms in the *aqmm* function^4^ are specified the same way as in other R linear mixed model fitting functions such as *lqmm ()* and *lme4 ()*. The output is separated into two parts: Parametric part that includes estimated fixed effects, with their standard errors (SE), in parentheses, and significant mixed effect representation of smoothing splines (see Table 3). Since the smooth coefficients are mostly uninterpretable, we focus on their variances to evaluate the spline coefficients' penalty at various quantiles (see Table 4 and Supplementary information). However, their estimated smoothed effects are depicted in Fig. 3. Table 4 also presents the estimated variance of the random effects from the fitted model (13).

**Table 3:** Parameter estimates followed by results of the smoothing terms from the AQMM for the CAPRISA 002 AI study data across different quantiles.

Fixed effects	$\widehat{Q}_{{0.05}}$ (SE)	$\widehat{Q}_{{0.25}}$ (SE)	$\widehat{Q}_{{0.5}}$ (SE)	$\widehat{Q}_{{0.75}}$ (SE)	$\widehat{Q}_{{0.95}}$ (SE)
Intercept	16.004 (0.6634)***	19.647 (0.4749)***	21.204 (0.5340)***	24.167 (1.0536)***	29.379 (0.6324) ***
Age	0.0398 (0.0156)**	0.0209 (0.0114)	0.0418 (0.0052)***	0.0331 (0.0078)***	0.0203 (0.0178)
Secondary school	− 0.4491 (0.5731)	− 0.4734 (0.4101)	− 0.0165 (0.6619)	0.0385 (1.0677)	0.8323 (0.5574)
Post HAART	0.7430 (0.0879)***	1.5296 (0.0598)***	1.5968 (0.0402)***	1.5292 (0.0546)***	1.7007 (0.1322)***
Baseline VL	− 3.83e−06 (8.42e−07)***	− 2.09e−06 (2.69e−07)***	− 1.79e−06 (2.41e−07)***	− 1.57e−06 (1.60e−07)***	− 1.70e−06 (2.21e−07)***
Urban	− 0.50002 (0.1668)**	0.2499 (0.0545)***	0.0998 (0.0334)**	0.1275 (0.1436)	− 0.8846 (0.2216)***
Stable partner	0.6135 (0.1655)***	0.3046 (0.1549)	0.5424 (0.1140)***	0.4907 (0.1594)**	0.6339 (0.2960)*
Many partners	− 2.2771 (0.2707)***	− 0.7858 (0.2589)**	− 0.8432 (0.1091)***	− 1.1719 (0.2569)***	− 3.6497 (0.4451)***
**Results of the smooth terms**					
s (Time)	− 2.5075 (0.5426)***	− 2.3766 (0.5549)***	− 2.1985 (0.4735)***	− 2.2829 (0.4999)***	− 2.3324 (0.4373)***
s (Baseline BMI)	5.4382 (1.0786)***	5.6868 (1.1094)***	5.5767 (1.3014)***	5.7904 (1.2077)***	5.2604 (1.0753)***

*Significance codes: 0 ‘***’, 0.001 ‘**’, 0.01 ‘*’, 0.05 ‘.’, 0.1 ‘ ’, 1. The reference categories are given in Table 2.

**Table 4:** Estimated variance of the random effects and smooth terms from the AQMM for the CAPRISA 002 AI study data.

Results across different quantiles							
	$\hat{Q}_{0.05}$	$\hat{Q}_{0.25}$	$\hat{Q}_{0.5}$	$\hat{Q}_{0.75}$	$\hat{Q}_{0.85}$	$\hat{Q}_{0.95}$	$\hat{Q}_{0.99}$
**Variance of the random effects**							
$\hat{\sigma }_{0}$(Intercept)	0.02748	0.8687	0.0354	0.2453	0.3454	0.0467	0.0033
$\hat{\sigma }_{0}$(Time)	8.104e−18	1.929e−16	3.328e−17	5.451e−17	7.671e−17	1.044e−17	2.963e−18
**Variance of the smooth terms**							
$\hat{\phi }_{Time}$	8.796	28.94	36.74	30.28	21.92	10.13	2.669
$\hat{\phi }_{Baseline BMI}$	1876.501	6463.83	7823.81	6290.32	4979.39	2183.69	576.902

According to Table 3, the age effect is positive and significant at the bottom, median, and at $\tau = 0.75$ quantile levels (see also Supplementary information). On the other hand, the effect of education on square root CD4 count does not seems to be significant across all quantiles after the patient had been initiated on HAART. The square root CD4 count across all quantiles is affected by post-HAART initiation as expected. A significant positive effect of HAART initiation on CD4 cell counts is observed at the median quantile and becomes roughly constant at higher quantiles (see Table 3 and Supplementary information). In addition, patients with stable sexual partners showed significant improvements in their CD4 cell count across all quantiles. The CD4 cell count is significantly lowered in patients who have many sexual partners, especially at the bottom ($\tau = 0.05$) and at the top ($\tau = 0.95, 0.99$) quantiles (Table 3).

Furthermore, we found a clear indication, at the bottom ($\tau = 0.05$) and more extreme quantiles ($\tau = 0.85, 0.95, 0.99$), that there is a significant negative effect of patients who were residing around the urban area on their CD4 cell count (see Table 3 and Supplementary information). Table 3 also shows that the negative effect of baseline viral load on the CD4 cell count is higher at the lower quantiles than at the median and higher quantiles (see also, Supplementary information). In addition, R package *aqmm()* sample outputs using CAPRISA 002 AI study data at $\tau = 0.25, 0.75, 0.85,$ and $0.99$ can be found in Supplementary information.

The variance of the first smooth term ($\hat{\phi }_{Time}$) indicates a stronger penalty on the spline coefficients at $\tau = 0.25, 0.5, 0.75, 0.85$ quantiles than at the bottom and at the top quantiles (Table 4). Similarly, the variance of the second smoother ($\hat{\phi }_{Baseline BMI}$) shows a strong penalty on the spline coefficients at $\tau = 0.25, 0.5, 0.75, 0.85$ quantiles than at the bottom and at more extreme quantiles. Table 4 shows that the random effects' variances have roughly constant variability of subject linear trends across the fitted quantiles (see, also, Supplementary information).

Based on the seven fitted quantile levels ($\tau = 0.05, 0.25, 0.5, 0.75, 0.85, 0.95, 0.99$), Fig. 3 depicts the two estimated smoothed covariate effects on patients’ CD4 counts. Patients enrolled in the CAPRISA 002 AI study exhibit nonlinear time effects on CD4 counts that are prominent at all quantile levels. As the quantile increases, its effect becomes stronger. However, it is after several treatment visits that such progress towards higher CD4 counts occurs. Consequently, the progression is slow until about 50 months, then it increases steadily thereafter across all quantile levels (Fig. 3).

Furthermore, overall fit quantile levels, the significant smoothed baseline BMI effect on patients' CD4 counts is roughly constant for patients with a baseline BMI of about 40 but gradually improves from there. Because of this, patients with low BMI need to be monitored carefully before and after HAART initiation. Despite this, physicians should not ignore patients with high BMI. According to our studies and other findings, a plausible explanation may be that BMI may affect drug metabolism and, thus, the progress of HAART and its immunological responses^20,67,68^. Moreover, higher levels of BMI have a more significant effect than lower levels (Fig. 3).

## Discussion and conclusion

As a cutting-edge statistical method for modeling percentiles of response variables conditioned on respective covariates, quantile regression is the most widely used. While regression for medians may be seen as more robust than regression for the mean, QR, a generalization of median regression, allows better exploration of data by allowing the modeling of conditional quantiles at low or high extents, such as the 5th and 95th percentiles. As a result, QR is becoming more common in clinical, biomedical, and other health-related research. Mean-based regression is used to formulate mixed-effects models and their estimated effects on the response variable. In some cases, this centrality-based inference method may not be the optimal method for dealing with the data since the data may not adequately represent their distribution. It has recently been demonstrated that QR has the potential to be extended to a mixed-effects modeling setting, even though QR was initially developed in a univariate setting^48,60,61^. Studies of quantile mixed-effects models have received increasing attention^15,48,60,61,69–76^.

Quantile mixed-effects models have been extended to additive models to obtain robust results across various quantile levels of the longitudinal outcome, which brings a rigorous covariates' effect^74–76^. The additive version of the quantile mixed-effects model has gained a great deal of popularity, as discussed above; because it offers an efficient and flexible framework for nonlinear and linear longitudinal forms of data analysis focused on features of the outcome beyond its central tendency^1,4,11,12,47,73,75,76^.

In this study, we investigated the effect of multivariate additive quantile mixed models of Geraci^4^ on the longitudinal CD4 count of HIV-infected patients across different quantile levels according to parametric and nonparametric covariate effects. By using this recently developed model, robust results are obtained, not only at the central location of the longitudinal outcome that may not be the best place to analyze the data but also at different points of the conditional distribution that gives an inclusive and more complete picture of the parametric as well as the nonparametric covariate effects.

A series of AQMM at $\tau = 0.05, 0.25, 0.5, 0.75, 0.85, 0.95$, and $0.99$ were performed, and the results were discussed. According to the results, patients' CD4 count is markedly increased after HAART initiation, and their baseline viral load shows a negative effect on the progression of their CD4 count over time, as we would have expected. All fitted quantiles of the response variable were affected by a significant nonlinear relationship between time and baseline BMI. Study results suggest that, across all fitted quantile levels, the patient's education level does not significantly influence the progression of CD4 counts over time. All but the most extreme quantiles of HIV-positive patients showed a significant difference in the CD4 count regardless of their age. In addition, CD4 cell recovery was found to be significant across all quantiles among patients with a stable sexual partner. Contrary to this, HIV-infected patients with many sexual partners during the treatment period showed a negative effect on CD4 cell count across all fitted quantile levels.

As we expected, the patient's CD4 count increased significantly after HAART was initiated, and their baseline viral load also showed a significant negative effect on the patient's CD4 count over time. Baseline BMI and time were also significant nonlinear effects in our analysis. Further, patients with higher BMIs at baseline have improved CD4 cell count over time after treatment. Despite this, higher BMI patients should not be ignored clinically. This study instead suggests that BMI can influence drug metabolism and, consequently, the immunological responses to HAART. According to the nonlinear time effect, patients' CD4 counts are not increasing rapidly over time. The growth starts after multiple treatment visits. Hence, the study suggests that HIV patients who are not clinically and immunologically stable on HAART could experience increased risks if exposed to COVID-19, especially if they are not on HAART immediately after HIV exposure.

One can estimate the covariate effects over the grid $\tau \in \left( {0, 1} \right)$ as per the analysis aspects. An investigator, however, should be cautious when using AQMM since the method needs some adjustment to control the estimation algorithm and demands more computing time to estimate the random effects^4^. For instance, for this study, it took 2–3 h to fit the proposed model (13) at a single $\tau$ as like Geraci^4^. To overcome this computational burden, Geraci^4^ suggested the necessity of further improvement to the AQMM. As the studied data set is an ongoing study, there is a plan to extend AQMM application to genetics in future work since it produces satisfactory results.

## Supplementary Information

Supplementary Information.

## Data Availability

The dataset used for this study can be obtained by requesting Dr. Nonhlanhla Yende-Zuma (Head of Biostatistics Unit, CAPRISA, Email: Nonhlanhla.Yende@caprisa.org) on reasonable request.

## Abbreviations

AMM: Additive mixed model
QR: Quantile regression
AQM: Additive quantile model
AQMM: Additive quantile mixed model
GAMLSS: Generalized additive model for location, scale, and shape
CAPRISA: Centre of the AIDS Programme of Research in South Africa
HIV: Human immunodeficiency virus
AIDS: Acquired immune deficiency syndrome
CD4: Cluster of difference 4 cell (t-lymphocyte cell)
VL: Viral load refers to the number of HIV copies in a milliliter of blood (copies/ml)
STD: Sexually transmitted diseases
ART: Antiretroviral therapy
ARV: Antiretroviral (drug)
HAART: Highly active antiretroviral therapy
WHO: World Health Organization
