# Supplementary material for: Additive quantile mixed effects modelling with application to longitudinal CD4 count data
Source: Sci Rep. 2021 Sep 9;11:17945. doi: 10.1038/s41598-021-97114-9 (PMC8429740; doi:10.1038/s41598-021-97114-9)
Supplement: Supplementary file 1 — Supplementary Information. [file 41598_2021_97114_MOESM1_ESM.pdf]

Additive Quantile Mixed Effects Modelling with Application to Longitudinal CD4 Count Data

Ashenafi A Yirga[1*], Sileshi F Melesse[1], Henry G Mwambi[1] and Dawit G Ayele[2]

1. School of Mathematics, Statistics, and Computer Science, University of KwaZulu-Natal, Pietermaritzburg, Private Bag X01, Scottsville, 3209, South Africa.
2. Institute of Human Virology, University of Maryland, School of Medicine, USA.

*Corresponding Author: Ashenafi Argaw Yirga, Ph.D. Candidate,
University of KwaZulu-Natal,
Pietermaritzburg, South Africa.
Email: ashu3argaw@gmail.com, or 216065934@stu.ukzn.ac.za

Supplementary information 1: R package additive quantile mixed model, *aqmm()*, sample outputs using CAPRISA 002 Acute Infection Study data across various quantile levels.

```
Quantile 0.05

Fixed effects:
                                    Estimate  Std. Error t value  Pr(>|t|)
(Intercept)                       1.6004e+01  6.6336e-01 24.1254 < 2.2e-16 ***
Age                               3.9817e-02  1.3558e-02  2.9369  0.003706 **
Educational_levelSecondary school -4.4909e-01  5.7315e-01 -0.7835  0.434240
ARTpost ART initiation            7.4305e-01  8.7964e-02  8.4472 6.219e-15 ***
Baseline_VL1                     -3.8346e-06  8.4209e-07 -4.5537 9.174e-06 ***
Residence_mod2                   -5.0002e-01  1.6685e-01 -2.9967  0.003076 **
Marital_statusStable              6.1352e-01  1.6551e-01  3.7068  0.000272 ***
Marital_statusMany               -2.2771e+00  2.7072e-01 -8.4112 7.802e-15 ***
s(Time_in_Months)Fx1             -2.5075e+00  5.4263e-01 -4.6210 6.854e-06 ***
s(Baseline_BMI1)Fx1               5.4382e+00  1.0786e+00  5.0419 1.034e-06 ***
---
Signif. codes:  0 ‘***’ 0.001 ‘**’ 0.01 ‘*’ 0.05 ‘.’ 0.1 ‘ ’ 1

Covariance matrix of the random effects:
               (Intercept) Time_in_Months
(Intercept)    2.748e-02   0.000e+00
Time_in_Months 0.000e+00   8.104e-18

Variances of the (random) smooth terms:
s(Time_in_Months)  s(Baseline_BMI1)
            8.796          1876.501

Residual scale parameter:  0.4223
Log-likelihood: -24280
Tuning parameter: 0.0001923

Number of observations: 7019
Number of groups: 235
```

```
Quantile 0.25

Fixed effects:
                                     Estimate  Std. Error t value  Pr(>|t|)
(Intercept)                        1.9647e+01  4.7491e-01 41.3708 < 2.2e-16 ***
Age                                2.0960e-02  1.1454e-02  1.8299  0.068760 .
Educational_levelSecondary school -4.7338e-01  4.1009e-01 -1.1543  0.249745
ARTpost ART initiation             1.5296e+00  5.9779e-02 25.5868 < 2.2e-16 ***
Baseline_VL1                      -2.0864e-06  2.6911e-07 -7.7530 4.538e-13 ***
Residence_mod2                     2.4989e-01  5.4473e-02  4.5875 7.927e-06 ***
Marital_statusStable               3.0457e-01  1.5489e-01  1.9664  0.050647 .
Marital_statusMany                -7.8584e-01  2.5890e-01 -3.0353  0.002724 **
s(Time_in_Months)Fx1              -2.3766e+00  5.5497e-01 -4.2824 2.875e-05 ***
s(Baseline_BMI1)Fx1                5.6868e+00  1.1094e+00  5.1261 6.982e-07 ***
---
Signif. codes:  0 ‘***’ 0.001 ‘**’ 0.01 ‘*’ 0.05 ‘.’ 0.1 ‘ ’ 1

Covariance matrix of the random effects:
               (Intercept) Time_in_Months
(Intercept)    8.687e-01   0.000e+00
Time_in_Months 0.000e+00   1.929e-16

Variances of the (random) smooth terms:
s(Time_in_Months)  s(Baseline_BMI1)
            28.94           6463.83

Residual scale parameter:  1.335
Log-likelihood: -21031
Tuning parameter: 0.0003077

Number of observations: 7019
Number of groups: 235
```

```
Quantile 0.5

Fixed effects:
                                   Estimate  Std. Error t value  Pr(>|t|)
(Intercept)                       2.1204e+01  5.3400e-01 39.7075 < 2.2e-16 ***
Age                               4.1792e-02  5.2561e-03  7.9511 1.358e-13 ***
Educational_levelSecondary school -1.6529e-02  6.6199e-01 -0.0250  0.980105
ARTpost ART initiation            1.5968e+00  4.0196e-02 39.7245 < 2.2e-16 ***
Baseline_VL1                     -1.7910e-06  2.4096e-07 -7.4325 3.088e-12 ***
Residence_mod2                    9.9844e-02  3.3382e-02  2.9910  0.003132 **
Marital_statusStable              5.4241e-01  1.1403e-01  4.7568 3.772e-06 ***
Marital_statusMany               -8.4317e-01  1.0906e-01 -7.7310 5.181e-13 ***
s(Time_in_Months)Fx1             -2.1985e+00  4.7348e-01 -4.6432 6.222e-06 ***
s(Baseline_BMI1)Fx1               5.5767e+00  1.3014e+00  4.2851 2.844e-05 ***
---
Signif. codes:  0 ‘***’ 0.001 ‘**’ 0.01 ‘*’ 0.05 ‘.’ 0.1 ‘ ’ 1

Covariance matrix of the random effects:
               (Intercept) Time_in_Months
(Intercept)    3.543e-02   0.000e+00
Time_in_Months 0.000e+00   3.328e-17

Variances of the (random) smooth terms:
s(Time_in_Months)  s(Baseline_BMI1)
            36.74           7823.81

Residual scale parameter:  1.735
Log-likelihood: -20671
Tuning parameter: 0.0003077

Number of observations: 7019
Number of groups: 235
```

```
Quantile 0.75

Fixed effects:
                                    Estimate  Std. Error t value  Pr(>|t|)
(Intercept)                       2.4167e+01  1.0536e+00 22.9374 < 2.2e-16 ***
Age                               3.3143e-02  7.7590e-03  4.2716 3.006e-05 ***
Educational_levelSecondary school  3.8519e-01  1.0677e+00  0.3608  0.718663
ARTpost ART initiation            1.5292e+00  5.4576e-02 28.0196 < 2.2e-16 ***
Baseline_VL1                     -1.5700e-06  1.6001e-07 -9.8123 < 2.2e-16 ***
Residence_mod2                    1.2747e-01  1.4362e-01  0.8876  0.375851
Marital_statusStable              4.9069e-01  1.5944e-01  3.0776  0.002381 **
Marital_statusMany               -1.1719e+00  2.5698e-01 -4.5603 8.916e-06 ***
s(Time_in_Months)Fx1             -2.2829e+00  4.9993e-01 -4.5665 8.680e-06 ***
s(Baseline_BMI1)Fx1               5.7904e+00  1.2077e+00  4.7945 3.188e-06 ***
---
Signif. codes:  0 ‘***’ 0.001 ‘**’ 0.01 ‘*’ 0.05 ‘.’ 0.1 ‘ ’ 1

Covariance matrix of the random effects:
               (Intercept) Time_in_Months
(Intercept)    2.453e-01   0.000e+00
Time_in_Months 0.000e+00   5.451e-17

Variances of the (random) smooth terms:
s(Time_in_Months)  s(Baseline_BMI1)
            30.28           6290.32

Residual scale parameter:  1.43
Log-likelihood: -21680
Tuning parameter: 0.0003077

Number of observations: 7019
Number of groups: 235
```

```
Quantile 0.85

Fixed effects:
                                    Estimate  Std. Error  t value  Pr(>|t|)
(Intercept)                       2.5845e+01  8.8147e-01  29.3207 < 2.2e-16 ***
Age                               3.1118e-02  1.2061e-02   2.5802 0.0105958 *
Educational_levelSecondary school  5.0743e-01  9.6045e-01   0.5283 0.5978665
ARTpost ART initiation            1.4546e+00  1.2778e-01  11.3838 < 2.2e-16 ***
Baseline_VL1                     -1.7132e-06  1.5806e-07 -10.8391 < 2.2e-16 ***
Residence_mod2                   -6.0903e-02  1.9103e-01  -0.3188 0.7501999
Marital_statusStable              6.2981e-01  1.3699e-01   4.5974 7.594e-06 ***
Marital_statusMany               -1.5711e+00  4.2531e-01  -3.6940 0.0002852 ***
s(Time_in_Months)Fx1             -2.2924e+00  3.8936e-01  -5.8877 1.642e-08 ***
s(Baseline_BMI1)Fx1               5.6964e+00  1.1277e+00   5.0513 9.898e-07 ***
---
Signif. codes:  0 ‘***’ 0.001 ‘**’ 0.01 ‘*’ 0.05 ‘.’ 0.1 ‘ ’ 1

Covariance matrix of the random effects:
               (Intercept) Time_in_Months
(Intercept)    3.454e-01   0.000e+00
Time_in_Months 0.000e+00   7.671e-17

Variances of the (random) smooth terms:
s(Time_in_Months)  s(Baseline_BMI1)
            21.92           4979.39

Residual scale parameter:  1.068
Log-likelihood: -22972
Tuning parameter: 0.0002564

Number of observations: 7019
Number of groups: 235
```

```
Quantile 0.95

Fixed effects:
                                    Estimate  Std. Error t value  Pr(>|t|)
(Intercept)                       2.9379e+01  6.3243e-01 46.4537 < 2.2e-16 ***
Age                               2.0274e-02  1.7881e-02  1.1338   0.25823
Educational_levelSecondary school  8.3235e-01  5.5746e-01  1.4931   0.13699
ARTpost ART initiation            1.7007e+00  1.3216e-01 12.8690 < 2.2e-16 ***
Baseline_VL1                     -1.7020e-06  2.2139e-07 -7.6881 6.714e-13 ***
Residence_mod2                   -8.8465e-01  2.2161e-01 -3.9920 9.212e-05 ***
Marital_statusStable              6.3398e-01  2.9600e-01  2.1418   0.03342 *
Marital_statusMany               -3.6497e+00  4.4508e-01 -8.2002 2.920e-14 ***
s(Time_in_Months)Fx1             -2.3324e+00  4.3732e-01 -5.3335 2.605e-07 ***
s(Baseline_BMI1)Fx1               5.2604e+00  1.0753e+00  4.8920 2.056e-06 ***
---
Signif. codes:  0 ‘***’ 0.001 ‘**’ 0.01 ‘*’ 0.05 ‘.’ 0.1 ‘ ’ 1

Covariance matrix of the random effects:
               (Intercept) Time_in_Months
(Intercept)    4.675e-02   0.000e+00
Time_in_Months 0.000e+00   1.044e-17

Variances of the (random) smooth terms:
s(Time_in_Months)  s(Baseline_BMI1)
            10.13           2183.69

Residual scale parameter:  0.4828
Log-likelihood: -26193
Tuning parameter: 0.0003846

Number of observations: 7019
Number of groups: 235
```

```
Quantile 0.99

Fixed effects:
                                     Estimate  Std. Error  t value  Pr(>|t|)
(Intercept)                        2.9479e+01  7.2187e-01  40.8373 < 2.2e-16 ***
Age                                3.5241e-02  3.2735e-02   1.0765 0.2829879
Educational_levelSecondary school  2.0311e+00  9.3341e-01   2.1760 0.0307302 *
ARTpost ART initiation             2.8819e+00  2.1028e-01  13.7050 < 2.2e-16 ***
Baseline_VL1                      -1.6168e-06  2.1655e-07  -7.4661 2.531e-12 ***
Residence_mod2                    -1.4498e+00  3.0687e-01  -4.7243 4.356e-06 ***
Marital_statusStable               2.8311e+00  1.8621e-01  15.2040 < 2.2e-16 ***
Marital_statusMany                -4.2155e+00  1.6546e-01 -25.4775 < 2.2e-16 ***
s(Time_in_Months)Fx1              -1.6920e+00  4.6815e-01  -3.6143 0.0003815 ***
s(Baseline_BMI1)Fx1                5.2328e+00  1.1256e+00   4.6487 6.075e-06 ***
---
Signif. codes:  0 ‘***’ 0.001 ‘**’ 0.01 ‘*’ 0.05 ‘.’ 0.1 ‘ ’ 1

Covariance matrix of the random effects:
               (Intercept) Time_in_Months
(Intercept)    3.326e-03   0.000e+00
Time_in_Months 0.000e+00   2.963e-18

Variances of the (random) smooth terms:
s(Time_in_Months)  s(Baseline_BMI1)
            2.669           576.902

Residual scale parameter:  0.1276
Log-likelihood: -30865
Tuning parameter: 0.0002404

Number of observations: 7019
Number of groups: 235
```
